# Service quality in decentralized community-based Covid-19 antigen rapid diagnostic testing programmes in the Federal Capital Territory, Nigeria

**DOI:** 10.1371/journal.pone.0310294

**Published:** 2024-12-10

**Authors:** Ekerette Udoh, Osayamen Okonkwo, Godpower Omoregie, Victoria Ura-Akubo, Nnamdi Anosike, Joshua Folorunsho, Evelyn Orakwelu, John Bimba, Karin Hatzold, Elizabeth L. Corbett, Emily Nightingale, Yasmin Dunkley

**Affiliations:** 1 Society for Family Health, Abuja, Nigeria; 2 Zankli Research Centre, Bingham University, Nasarawa, Nigeria; 3 Population Services International, Washington, DC, United States of America; 4 London School of Hygiene and Tropical Medicine, London, United Kingdom; University of Manitoba Faculty of Health Sciences, NIGERIA

## Abstract

**Introduction:**

Decentralized COVID-19 testing with antigen rapid diagnostic tests (Ag-RDT) is recommended by the Nigerian Centre for Disease Control for community-level services. These services have been provided in Primary Healthcare Centers, Community Pharmacies, and licensed “Patent Medicine Stores” that serve the least affluent communities. To support quality assurance, we applied an adapted version of SPI-RT (Stepwise Process for Improving the Quality of HIV Rapid and Recency Testing) to sites providing COVID-19-RDTs in Federal Capital Territory of Nigeria.

**Methods:**

Between September 2022 and February 2023 community healthcare facilities (48 Community Pharmacies, 21 Patent Medicine Stores, 79 Primary Health Centers) were evaluated using Stepwise Process for Improving the Quality of SARS-CoV-2 Antigen Rapid Diagnostic Testing (SPI-RT) Checklist, tailored to the local implementation context. Evaluated domains included service quality, documents and records, personnel training and certification, safety, physical infrastructure, pre-testing phase, testing phase, and post-testing phase. Each facility received an overall score, expressed as a percentage indicating their performance level.

**Results:**

41/79 (52%) of Primary Health Centers scored at least 90% for service quality, as did 19/48 (40%) of pharmacies, with only 1/21 (4.8%) Patent Medicine Store. Apart from personnel training and certification, Primary Health Centers scored highest across most domains of service quality, followed by Community Pharmacies. The lowest median score in any domain was in the Patent Medicine Stores on testing and safety at 60% for both post-testing phase and safety.

**Conclusion:**

Primary Healthcare Centers and Community Pharmacies can provide quality decentralized testing for COVID-19. Patent Medicine Stores may need additional support including monitoring and quality improvement initiatives to ensure the provision of high-quality decentralized COVID-19 rapid testing services.

## Introduction

Testing for SARS-CoV-2 played a critical role in the global response to the COVID-19 pandemic. Since the onset of the disease in Nigeria in February of 2020, the nation’s testing capability increased progressively because of concerted efforts by governments and partner organizations in scaling-up testing. The country adopted various testing strategies, including the use of antigen-based Rapid Diagnostic Tests (Ag-RDT), molecular polymerase chain reaction (PCR) tests, GeneXpert and genome sequencing for case detection and identification of emerging variants. Ag-RDT is a valuable screening tool for early diagnosis of asymptomatic and symptomatic infections, due to its short turn-around time and reduced cost [1,2]. Early diagnosis is a key factor in preventing community transmission for example by facilitating contact tracing and active monitoring [3,4].

In Nigeria, Ag-RDT was adopted as a strategy for screening and early diagnosis of COVID-19 and to scale-up testing for the virus following the release of a national guideline on its use in January 2021 [3,5]. Its introduction has allowed for expansion and decentralization of testing capacity, utilizing existing health structures and systems. Expansion of testing has resulted in increased uptake; the average daily COVID-19 tests conducted from all diagnostic procedures rose from 2,500 samples in April 2020, to 4,000 samples by December 2020, and 5,250 samples by April 2021 [6]. By December 18^th^, 2022, the Nigeria Centre for Disease Control (NCDC) reported a total of 5,812,382 cumulative tests from all diagnostic profiles, of which Ag-RDT constituted 1.8 million (31%) [7].

Programs to decentralize testing for COVID-19 involve task-shifting to community health workers, non-laboratory professionals and/or lay cadres [8]. In Nigeria, community-based decentralization included shifting testing responsibly to Primary Healthcare Workers, Community Pharmacists and Patent Medicine Vendors who often serve as the first point of contact for basic healthcare in the country [9,10]. These decentralization programs aimed to curb the spread of the virus by improving access to testing and diagnosis for those living in poorer communities, or otherwise under-served areas.

Community-based decentralization of COVID-19 testing can improve access and increase uptake of testing as well as strengthen and contribute to COVID-19 surveillance and response. However, risks of decentralized services include service quality; community facilities may not be subject to the same clinical standards as more established health services, buildings may have poorer infrastructure, with potentially lower-skilled, or less trained workers, fewer safety standards, lower adherence to quality standards in testing, reporting, and management of results, as well as poorer testing process, and data management [9,11,12].

To support quality assurance of a community-based decentralized testing model, this study evaluated COVID-19 testing sites in the Federal Capital Territory of Nigeria. The information learned from this study aims to guide optimization of decentralization of COVID19 AG-RDT in Nigeria and other countries where similar models are being implemented.

## Methods

### Setting

An assessment was conducted at Community Healthcare Facilities implementing scaled-up decentralized COVID19 Ag-RDT across six (6) area councils in the Federal Capital Territory (FCT) of Nigeria. Sites were selected through convenience for assessment, from a total of 222 sites set up in 2022 in the FCT through the Society for Family Health STAR 3ACP Project (funded by UNITAID through Population Services International). A total of 148 community healthcare facilities were assessed: including 48 Community Pharmacies, 21 Patent Medicine Stores and 79 Primary Healthcare Centers. The assessed facilities implemented COVID19 Ag-RDT using two country-approved professional test kits: Standard Q SD Biosensor and Abbot Panbio.

### Data collection and analysis

The assessment was conducted using a modified Stepwise Process for Improving the Quality of SARS-CoV-2 Antigen Rapid Diagnostic Testing (SPI-RT) Checklist. SPI-RT Checklist was developed by the African Society for Laboratory Medicine, the African Union, and Africa CDC for use by program supervisors, implementers, mentors, auditors undertaking testing site assessments to support quality assurance to ensure the optimal use of COVID19 Ag-RDT in various settings, including Community Healthcare Facilities [13]. The SPI-RT checklist has 8 domains: Documents and Records, Personnel Training and Certification, Safety, Physical Infrastructure, Pre-Testing Phases, Testing Phase, Post-Testing Phase, and External Quality Assessment (EQA), encompassing activities related to service provision, documentation for services, preparation for sample collection, sample collection, testing, result interpretation, and reporting of the test result. In this evaluation, we assessed 7 of the 8 domains of service quality removing the section that assessed external quality audit (EQA) of facilities, because in the program the facilities were not enrolled for EQA, which involves sharing of samples with a national reference laboratory, sample retesting using other laboratories, and conducting sample storage. We also removed 3 questions from the documentation section and 2 from the safety domain as these were not relevant to the level of provision at these community facilities. However, we incorporated an additional 6 questions in the SPI-RT tool including a reliability assessment of facility-level reported data and how well commodities were documented within the Document and Records, and Post-Testing domains (**S1 Table**).

Data collection involved supervision visits to the testing sites from trained researchers, government officials, and non-governmental implementers. The evaluators were trained on how to use the SPI-RT tool and data collection performed using KoboCollect hosted on android digital mobile devices [14]. Clients were mobilized to attend sites for observation and evaluation of the testing process. Upon completion of the assessment, verbal corrective actions were made for identified deficiencies/areas for improvement/non-conformances of the testing sites. Data collection for this assessment started on the 27-09-2022 and ended on 22-02-2023.

In the modified SPI-RT checklist there were a total of 69 questions or assessment items, with each of three possible responses corresponding to a numeric score as follows: Items marked as “Yes” received a score of one point; items marked "Partial" received 0.5 points; and items marked "No" received zero points. For each domain, the total points scored by the testing site were summed and expressed as a percentage of that domain. The overall score for the testing facility was expressed as the average across all domains.

The average percentage score was classified to correspond to a certain performance level as recommended in the SPI-RT checklist [13]. Overall scores less than 40% (Level 0) indicated that the testing facility needs improvement in all areas and immediate remediation for COVID19 Ag-RDT. Scores from 40% - 59% (Level 1) indicated the testing facility needs improvement in specific areas for COVID19 Ag-RDT; scores from 60% - 79% (Level 2) indicated that the testing facility partially meets minimum quality requirements for COVID19 Ag-RDT; scores of 80% - 89% (Level 3) implied that the testing facility was closer to meeting the quality requirements for COVID19 Ag-RDT; while scores of 90% - 100% (Level 4) indicated that the testing facility had fully met all quality requirements for COVID19 Ag-RDT. Descriptive statistics were used to summarize the distribution of performance scores for each facility type using percent, median and standard interquartile range, while differences in quality performance between facilities were tested using Kruskal-Wallis non-parametric test, with Bonferroni-corrected Mann Whitney U-test to identify pairwise differences between facility types.

### Ethics

As decentralized COVID19 Ag-RDT was national policy, and research was conducted as part of an assessment of service quality, consent waivers was obtained from ethics boards in lieu of written informed consent. Ethical approval was received as part of the study “3ACP-Nigeria: Enhancing access to COVID-19 rapid tests and self-testing” by the Federal Capital Territory Health Research Ethics Committee (FHREC), approval number FHREC/2022/01/29/09-03-22, WHO ERC (CERC.0165), and the London School of Hygiene and Tropical Medicine (26886).

## Results

Of 148 surveyed facilities, Primary Healthcare Centers made up over half of assessed facilities (53%, 79/148), Community Pharmacies constituted a third of assessed facilities (32%, 48/148), followed by Patent Medicine Stores (14%, 21/148). Two of the facilities (1.4%, 2/148) scored less than 40 thus placed at level ‘0’ on the quality performance rating, both Community Pharmacies. Fifteen facilities (10.1%, 15/148) scored between 40 and 59, thus places at level ‘1’ on the rating, with four Community Pharmacies and nine Patent Medicine Stores falling into this category (**Table 1**).

**Table 1:** Facility average quality performance rating.

Characteristic	Overall, N = 148^1^	Community pharmacy, N = 48^1^	Patent Medicine Store, N = 21^1^	Primary healthcare centre, N = 79^1^
**Performance quality rating**				
Level 0: Less than 40%	2 (1.4%)	2 (4.2%)	0 (0%)	0 (0%)
Level 1: 40% - 59%	15 (10.1%)	4 (8.3%)	9 (43%)	2 (2.5%)
Level 2: 60%-79%	27 (18.2%)	6 (12%)	8 (38%)	13 (16%)
Level 3: 80%-89%	43 (29.1%)	17 (35%)	3 (14%)	23 (29%)
Level 4: 90%-100%	61 (41.2%)	19 (40%)	1 (4.8%)	41 (52%)

^1^n (%).

Most facilities scored above 80 on the quality performance rating–corresponding to at least a level 3 on the quality rating—with Primary Healthcare Centers being the most consistent performers in terms of quality. Community Pharmacies and Patent Medicine Stores showed variability in quality performance, with 15 facilities falling below 60 (level 2).

**Fig 1:**
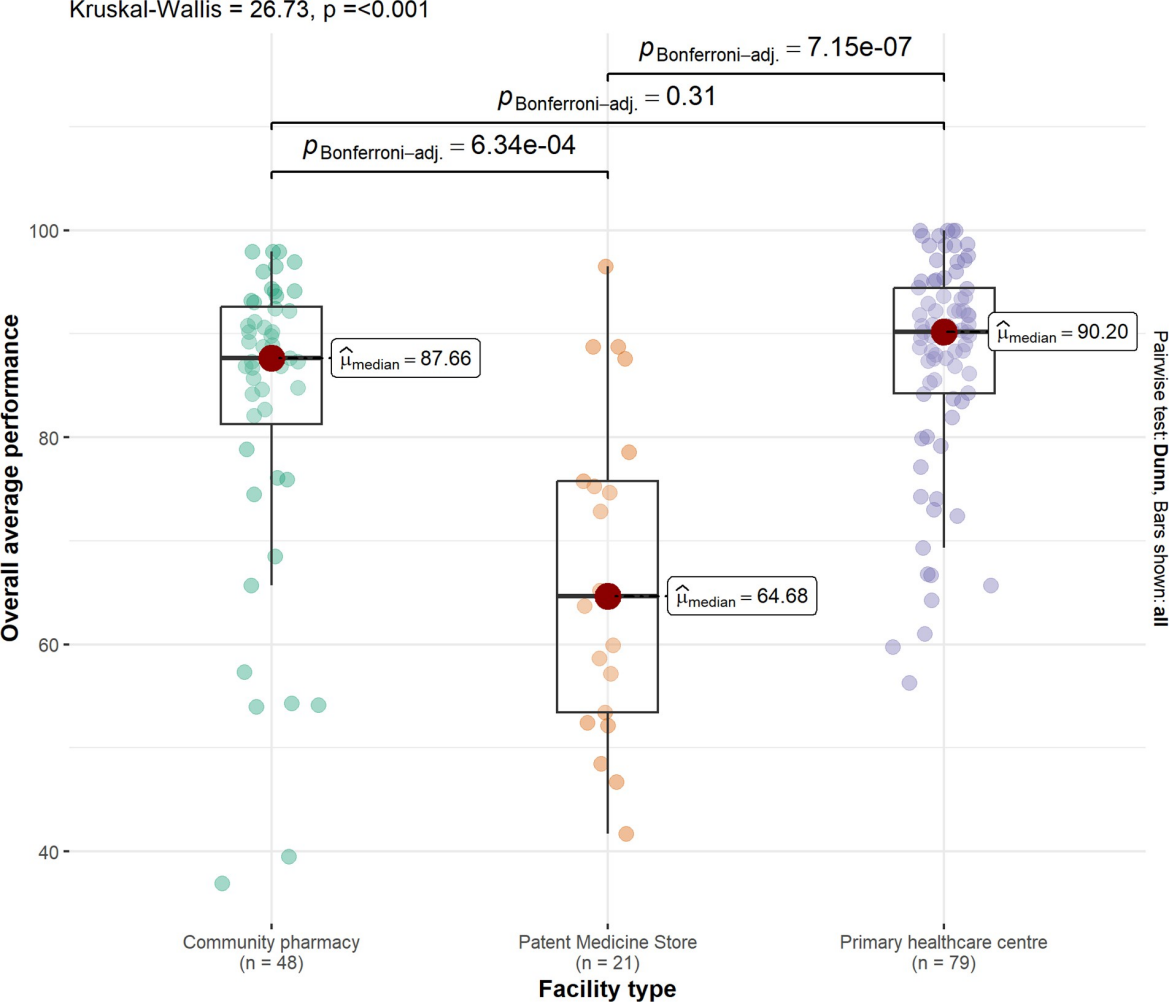
Comparison of COVID-19 RDT service quality performance across health facilities. Community health facility differences in performance in service quality for COVID-19 RDT. Median scores by facility type are indicated by red dots, alongside upper and lower quartiles. Kruskal-Wallis non-parametric test was used to compare the medians across all facility types. Bonferroni-corrected Mann Whitney U-test p-values for pairwise comparisons between facility types are presented above.

There was strong evidence that Primary Healthcare Centers (median score: 90.2) and Community Pharmacies (83.6) performed better than Patent Medicine Stores (64.6), (p<0.001 for both pairwise comparisons). However, there was no evidence of a difference in quality between Primary Healthcare Centers and Community Pharmacies (p = 0.31) (**Fig 1**).

Within individual domains, Primary Healthcare Centers scored higher in most of these, with physical infrastructure and safety being the domains where they performed better than Community Pharmacies and Patent Medicine Stores (**Table 2**).

**Table 2:** Comparison of COVID-19 RDT quality domains across health facilities.

Characteristic	Overall, N = 148	Community pharmacy, N = 48	Patent Medicine Store, N = 21	Primary healthcare centre, N = 79	p-value^1^
**Documents and records**					0.002
*Median (IQR)*	82 (67, 93)	84 (64, 97)	64 (54, 79)	86 (75, 93)	
**Personnel training and certification**					0.5
*Median (IQR)*	100 (90, 100)	100 (90, 100)	100 (100, 100)	100 (80, 100)	
**Physical infrastructure**					<0.001
*Median (IQR)*	86 (71, 100)	86 (71, 86)	71 (57, 79)	100 (86, 100)	
**Safety**					<0.001
*Median (IQR)*	90 (75, 100)	90 (79, 100)	60 (40, 70)	100 (85, 100)	
**Pre-testing phase**					0.013
*Median (IQR)*	80 (80, 100)	90 (80, 100)	80 (60, 80)	80 (80, 100)	
**Testing phase**					0.002
*Median (IQR)*	97 (81, 100)	97 (81, 100)	66 (0, 97)	100 (88, 100)	
**Post-testing phase**					0.001
*Median (IQR)*	80 (70, 100)	80 (78, 100)	60 (50, 80)	80 (80, 100)	
**Overall percent**					<0.001
*Median (IQR)*	88 (76, 93)	88 (81, 93)	65 (53, 76)	90 (84, 94)	

^1^Kruskal-Wallis rank sum test.

The testing phase showed variations across facilities, with Primary Healthcare Centers performing better than the other two types of facilities. Primary Healthcare Centers had the highest median scores for most domains, with a median score of 86 (IQR: 75, 93), for documentation and report, 100 (IRQ: 86, 100) physical infrastructure, and 100 (IRQ: 85, 100) for safety. The median scores for Community Pharmacies and Patent Medicine Stores were lower, with Patent Medicine Stores having the lowest median score of 60 for both post-testing phase and safety.

## Discussion

Primary Healthcare Centers are the most consistent performers in terms of quality of COVID-19 Ag-RDT. Primary Healthcare Centers also perform better at documentation and records, physical infrastructure, and safety compared to Community Pharmacies and Patent Medicine Stores. This is encouraging as this level of health facility is critical in health service delivery in Nigeria, essential to the provision of basic and comprehensive health services including disease prevention and control [15].

A previous study in Ekiti, Nigeria, showed lower COVID-19 RDT performance quality scores at secondary and tertiary facilities than at Primary Healthcare Centers. This is unexpected as higher-level facilities are usually considered to have better infrastructure [16]. The higher performance scores in our study could be explained by the extensive support facilities received in the intervention program. However, the modification of the evaluation tool limits the comparability of the results. The convenience sampling strategy also limits external validity, as those community facilities that were available and responded during our assessment, may have been those more likely to perform well in the assessment.

Community Pharmacies and Patent Medicine Stores showed reasonably good quality of COVID-19 RDT testing service, although some sites performed poorly in certain domains. These finding are important as community facilities such as pharmacies and Patent Medicine Stores are often the first level of contact for healthcare services, especially in rural areas where access to Primary Healthcare Centers is limited [17,18]. Community Pharmacies and Patent Medicine Stores have potential to provide quality COVID-19 RDT testing services, there is need for improvement of quality performance to ensure that they meet the required standards. The better performance by primary healthcare facilities is expected as they have better physical infrastructure and safety standards [19]. Additionally, these centers have more resources, medical exposure and training [19]. However, previous research indicates the performance of Patent Medicine Stores can be improved with appropriate training and support [18].

Recommendations for effective and adequate decentralization of COVID-19 testing at community level include provisions for comprehensive training and supervision on safe collection, handling of samples, testing and interpretation of test results [8]. Additionally, adequate, and appropriate biosafety measures including waste management and infection prevention and control (IPC) measures should be put in place. Engagement in quality assurance activities, which can include site supervision and mentorship is also recommended [8].

## Conclusion

Our study provides important insights into the quality of COVID-19 Ag-RDT testing delivered at community facilities in Nigeria as part of a decentralized testing strategy. Primary Healthcare Centers and Community Pharmacies can provide quality decentralized testing for COVID-19, while delivery through Patent Medicine Stores may require additional support to improve. The quality of COVID-19 Ag-RDT across health facilities, particularly in Patent Medicine Stores, is critical in ensuring accurate diagnosis, effective treatment, and prevention of the spread of the virus. Training and support such as monitoring, and quality improvement initiatives can improve the performance of community facilities to ensure equitable access to high quality decentralized services for COVID-19 testing.

## Supporting information

S1 Table Stepwise Process for Improving the Quality of SARS-CoV-2 Antigen Rapid Diagnostic Testing (SPI-RT) checklist. (DOCX)

S1 File (DOCX)
