# Supplementary material for: Service quality in decentralized community-based Covid-19 antigen rapid diagnostic testing programmes in the Federal Capital Territory, Nigeria
Source: PLoS One. 2024 Dec 10;19(12):e0310294. doi: 10.1371/journal.pone.0310294 (PMC11630617; doi:10.1371/journal.pone.0310294)
Supplement: S1 Table — (DOCX) [file pone.0310294.s001.docx]

**S1 Table. Stepwise Process for Improving the Quality of SARS-CoV-2 Antigen Rapid Diagnostic Testing (SPI-RT) Checklist.**

**TESTING FACILITY COMPONENTS FOR ASSESSMENT**

For each of the sections listed below, please indicate a response **Yes, Partial or No** against each requirement/question. Indicate “**Yes**” only when all elements are satisfactorily present. Provide comments for each “**Partial**” or “**No**” response. For each ‘Yes’ response, award a score of 1 point in the adjacent cell, for Partial award 0.5 point and for No, a ‘0’ point score is awarded. State N/A in the comments section if “not applicable” where appropriate (*).

|  | **DOCUMENTATION AND RECORDS** | YES | PARTIAL | NO |
| --- | --- | --- | --- | --- |
| 1.1 | Are the following guidelines specific for SARS-CoV-2 Antigen Rapid Diagnostic testing available at the testing facility?  ***Instruction:*** *The Evaluator / Auditor / Mentor / Supervisor must establish and determine the relevant latest international & national guidelines before assessing the testing facility.*  *For each guideline(s) listed below, award 1 point if the current version of the guideline is available, award 0 point if unavailable (and 0.5 points if available but not current version or otherwise)* |  |  |  |
| *a.* | *Are there SOPs and/or job aides in place to implement safety practices?* |  |  |  |
| *b.* | *Are there SOPs and/or job aides in place on how to dispose of infectious and non-infectious waste?* |  |  |  |
| *c.* | *Are there SOPs and/or job aides in place to manage spills of COVID-19 samples, blood and other body fluids?* |  |  |  |
| 1.2 | Is the national SARS-CoV-2 Antigen Rapid Diagnostic testing algorithm available at the testing facility/site? |  |  |  |
| 1.3 | Are there SOPs and/or job aides in place describing how client information should be recorded in the national SARS-CoV-2 Antigen Rapid Diagnostic testing database? |  |  |  |
| 1.4 | For each of the COVID19 Ag-RDT kit in use at the testing facility, are the manufacturer instructions/ manuals/ inserts available and accessible to testers? |  |  |  |
| 1.5 | Are SOPs and/or job aides in place for each SARS-CoV-2 Antigen Rapid Diagnostic rapid test used in the testing algorithm available and posted at the testing point? |  |  |  |
| 1.6 | Are national biosafety guidelines for COVID-19 infection prevention and control available and accessible to the testers? |  |  |  |
| 1.7 | Is there a national standardized SARS-CoV-2 Antigen RDT tests register/logbook available for recording test results? |  |  |  |
| 1.8 | Are there records indicating all testers have demonstrated competency in SARS-CoV-2 Antigen Rapid Diagnostic testing prior to client testing? |  |  |  |
| 1.9 | Does data reported in the facility register match data reported on the ECIF platform?* |  |  |  |
| 1.10 | Does data reported in the facility register match data reported to the program?* |  |  |  |
| 1.11 | Does data reported on the ECIF platform match data reported to the program?* |  |  |  |
| 1.12 | Are stock cards available and completely filled appropriately?* |  |  |  |
| 1.13 | Are client data in the facility register filled completely and appropriately?* |  |  |  |

| 1. **PERSONNEL TRAINING AND CERTIFICATION** | | YES | PARTIAL | NO |
| --- | --- | --- | --- | --- |
| 2.1 | Have all testers received training on the procedure for COVID-19 Ag Rapid testing *(including sample collection and testing)*? |  |  |  |
| 2.2 | Are the testers trained on the use of standardized SARS-CoV-2 Antigen Rapid Diagnostic Test registers/logbooks/electronic case investigation form? |  |  |  |
| 2.3 | Are the testers trained on quality control (QC) processes? |  |  |  |
| 2.4 | Are the testers trained on safety, risk assessment and waste management procedures and practices? |  |  |  |
| 2.5 | Are only certified testers allowed to perform SARS-CoV-2 Antigen Rapid Diagnostic Test? |  |  |  |

|  | **PHYSICAL FACILITY** | YES | PARTIAL | NO |
| --- | --- | --- | --- | --- |
|  | Is there a designated area for SARS-CoV-2 Antigen Rapid Diagnostic testing? |  |  |  |
|  | Is the testing area/facility clean and organized for SARS-CoV-2 Antigen Rapid Diagnostic testing? |  |  |  |
|  | Is sufficient lighting available in the designated testing area? |  |  |  |
|  | Are the COVID19 Ag-RDTs kits stored within temperature range based on the manufacturers’ instructions? |  |  |  |
|  | Is there sufficient and secure storage space for test kits and other consumables? |  |  |  |
|  | Is the space allocated for SARS-CoV-2 Antigen Rapid test testing adequate to perform the work without compromising the quality and safety of patients and testing personnel/health workers? |  |  |  |
|  | Is the COVID-19 sample collection area separated from the patient examination/testing areas/room(s)? |  |  |  |

| 1. **SAFETY** | | YES | PARTIAL | NO |
| --- | --- | --- | --- | --- |
|  | Are the following personal protective equipment available in sufficient quantity at the testing facility? ***Instruction:*** *For each of the listed PPE below, award 1 point if available insufficient quantities, 0 point if unavailable and 0.5 point if available but in insufficient quantities* |  |  |  |
| *a.* | *Gloves* |  |  |  |
| *b.* | *Gowns/Laboratory Coats* |  |  |  |
| *c.* | *Face masks* |  |  |  |
|  | Is PPE consistently and properly used by all testers through the testing process? ***Instructions:*** *The evaluator/assessor/supervisor observes for consistent use of the PPE throughout the processes before scoring this requirement* |  |  |  |
|  | Is there an installation/sink/facility dedicated to hand washing? |  |  |  |
|  | Is there clean water and soap available for hand washing? |  |  |  |
|  | Is an appropriate disinfectant to clean the work area available? |  |  |  |
|  | Are leak-proof biohazard bags and appropriate waste bins available and properly used on the testing site? |  |  |  |
|  | Are sharps, infectious, and non-infectious waste handled properly? |  |  |  |
|  | Are infectious and non-infectious waste containers emptied regularly per the SOP and/or job aides? |  |  |  |

| 1. **PRE-TESTING PHASE** | | YES | PARTIAL | NO |
| --- | --- | --- | --- | --- |
|  | Is the national SARS-CoV-2 Antigen Rapid Diagnostic testing algorithm being used at the testing facility/point and adhered to? |  |  |  |
|  | Is there a process in place for an alternative SARS-CoV-2 Antigen Rapid Diagnostic testing algorithm in case of expired or shortage of test kit(s)? |  |  |  |
|  | Are all the test kits currently in use within the expiration date? |  |  |  |
|  | Are test kits labeled with date received and initials? |  |  |  |
|  | Are there sufficient supplies available for client sample collection? |  |  |  |
|  | Are client identifiers recorded in the SARS-CoV-2 Antigen Rapid Diagnostic testing register per national guidelines and on test devices? |  |  |  |
|  | Are client data entered on the ECIF platform with verification code generated? |  |  |  |

| 1. **TESTING PHASE** | | YES | PARTIAL | NO |
| --- | --- | --- | --- | --- |
|  | Are timers available and used routinely for SARS-CoV-2 Antigen Rapid Diagnostic testing? |  |  |  |
|  | Did the tester put on the appropriate PPE for testing? |  |  |  |
|  | Are sample collection devices used correctly? |  |  |  |
|  | Are testing procedures adequately followed? |  |  |  |
|  | IQC performed and documented according to national/international guidelines or kit manufacturer instructions? |  |  |  |
|  | Did the tester adhere to the manufacturer instructions for using the SARS-CoV-2 Antigen RDT? |  |  |  |
|  | Did the tester have all the necessary supplies to perform the SARS-CoV-2 Antigen RDT procedure before starting the sample testing process? |  |  |  |
|  | Did the tester set up the workstation correctly? |  |  |  |
|  | Did the tester check the expiry date of the SARS-CoV-2 Antigen RDT? |  |  |  |
|  | Did the tester check that the test device and the desiccant pack in the foil pouch were not damaged or invalid? |  |  |  |
|  | Did the tester insert the swab into an extraction buﬀer tube and, while squeezing the buﬀer tube, stir the swab? |  |  |  |
|  | Did the tester remove the swab while squeezing the sides of the tube to extract the liquid from the swab? |  |  |  |
|  | Did the tester press the nozzle cap tightly onto the tube? |  |  |  |
|  | Did the tester apply the required number of drops of extracted specimen to the specimen well of the test device? |  |  |  |
|  | Are incorrect/invalid QC results properly recorded? |  |  |  |
|  | Are appropriate steps taken and documented when QC results are incorrect and/or invalid? |  |  |  |

| 1. **POST TESTING PHASE** | | YES | PARTIAL | NO |
| --- | --- | --- | --- | --- |
|  | Is the national standardized register/logbook being used correctly and consistently to record SARS-CoV-2 Antigen RDT tests results? |  |  |  |
|  | Are all the elements in the register/ logbook recorded/captured correctly? (e.g., client demographics, kit names, lot numbers, expiration dates, tester name, individual and final SARS-CoV-2 Antigen RDT tests results, etc.)? |  |  |  |
|  | Is there documented evidence that clinicians are immediately notified of SARS-CoV-2 test positive results to inform timely patient isolation and management/treatment? |  |  |  |
|  | Are invalid test results recorded in the register/logbook as well? |  |  |  |
|  | Are invalid tests repeated and results properly recorded in the register/logbook? |  |  |  |
|  | Are all client documents and records securely kept throughout all phases of the testing process? |  |  |  |
|  | Are all registers/logbooks and other documents kept in a secure location when not in use? |  |  |  |
|  | Are registers/logbooks properly labeled and archived when full? |  |  |  |
|  | Are results entered on the ECIF platform and data entry finalized?* |  |  |  |

* Questions added to the SPI-RT Checklist to assess reliability of facility-level reporting and documentation of commodities.
